# Sleep quality, autonomic dysfunction and renal function in diabetic patients with pre-CKD phase

**DOI:** 10.1038/s41598-021-98505-8

**Published:** 2021-09-24

**Authors:** Manabu Kadoya, Akiko Morimoto, Akio Miyoshi, Miki Kakutani-Hatayama, Kae Kosaka-Hamamoto, Kosuke Konishi, Yoshiki Kusunoki, Takuhito Shoji, Hidenori Koyama

**Affiliations:** Department of Diabetes, Endocrinology and Clinical Immunology, Hyogo College of Medicine, 1-1 Mukogawa-cho, Nishinomiya, Hyogo 663-8501 Japan

**Keywords:** Endocrinology, Health care, Health occupations, Medical research, Nephrology

## Abstract

Diabetes has been established as a strong risk factor for chronic kidney disease (CKD). Sleep apnea, poor sleep quality (PSQ), and autonomic imbalance are also considered to be potential risk factors for decline in renal function, though no known study has examined their integrated predictive value in diabetic and non-diabetic patients without CKD. The present cohort consisted of 754 serial patients (diabetes; n = 231, non-diabetes; n = 523) without CKD registered in the Hyogo Sleep Cardio-Autonomic Atherosclerosis (HSCAA) study. Patients underwent examinations to determine respiratory event index and objective sleep quality using actigraphy, as well as heart rate variability (HRV). Renal outcome was defined as a decline in estimated glomerular filtration rate to less than 60 ml/min/1.73 m^2^ for more than 3 months. Kaplan–Meier analysis showed that diabetic patients with PSQ or low HRV, but not sleep apnea, had a significantly increased risk for renal outcome. Furthermore, Cox proportional hazards analysis revealed that PSQ was significantly associated with elevated risk of renal outcome (HR: 2.57; 95% CI: 1.01–6.53, *p* = 0.045) independent of sleep apnea and classical risk factors. Low HRV tended to be, but not significantly (*p* = 0.065), associated with the outcome. In non-diabetic patients, PSQ was also significantly and independently associated with renal outcome, whereas sleep apnea and low HRV were not. In conclusion, PSQ and low HRV appear to be important predictors of decline in renal function in diabetic patients without CKD.

## Introduction

Diabetes mellitus (DM) has become established as one of the major risk factors for reduced renal function and chronic kidney disease (CKD)^1^, while it is also known to be associated with glycemic abnormalities, which cause microvascular complications. Additionally, recent studies have noted that sleep problems, including obstructive sleep apnea and poor sleep quality (PSQ), are becoming increasingly recognized as important risk factors for progression of CKD and changes in estimated glomerular filtration rate (eGFR)^2–5^. Moreover, autonomic nervous function, which regulates the global health of the vasculature, such as hemodynamics, vascular tone, metabolism, and inflammation, has also been shown to be associated with progression of renal dysfunction and CKD-related hospitalization^6^. Autonomic imbalance has been proposed to be an important mechanistic factor underlying the association of sleep quality with renal function^7–9^, though their integrated mutual significance remains unknown. Of particular importance, the impact on renal function in diabetic as compared with non-diabetic patients has yet to be elucidated.

Using data obtained in the Hyogo Sleep Cardio-Autonomic Atherosclerosis (HSCAA) cohort study, the aim of the present prospective investigation was to examine and compare the mutual integrated impact of sleep apnea, PSQ, and autonomic dysfunction on future decline in renal function in diabetic and non-diabetic patients without CKD.

## Methods

### Study design and participants

**Figure 1:**
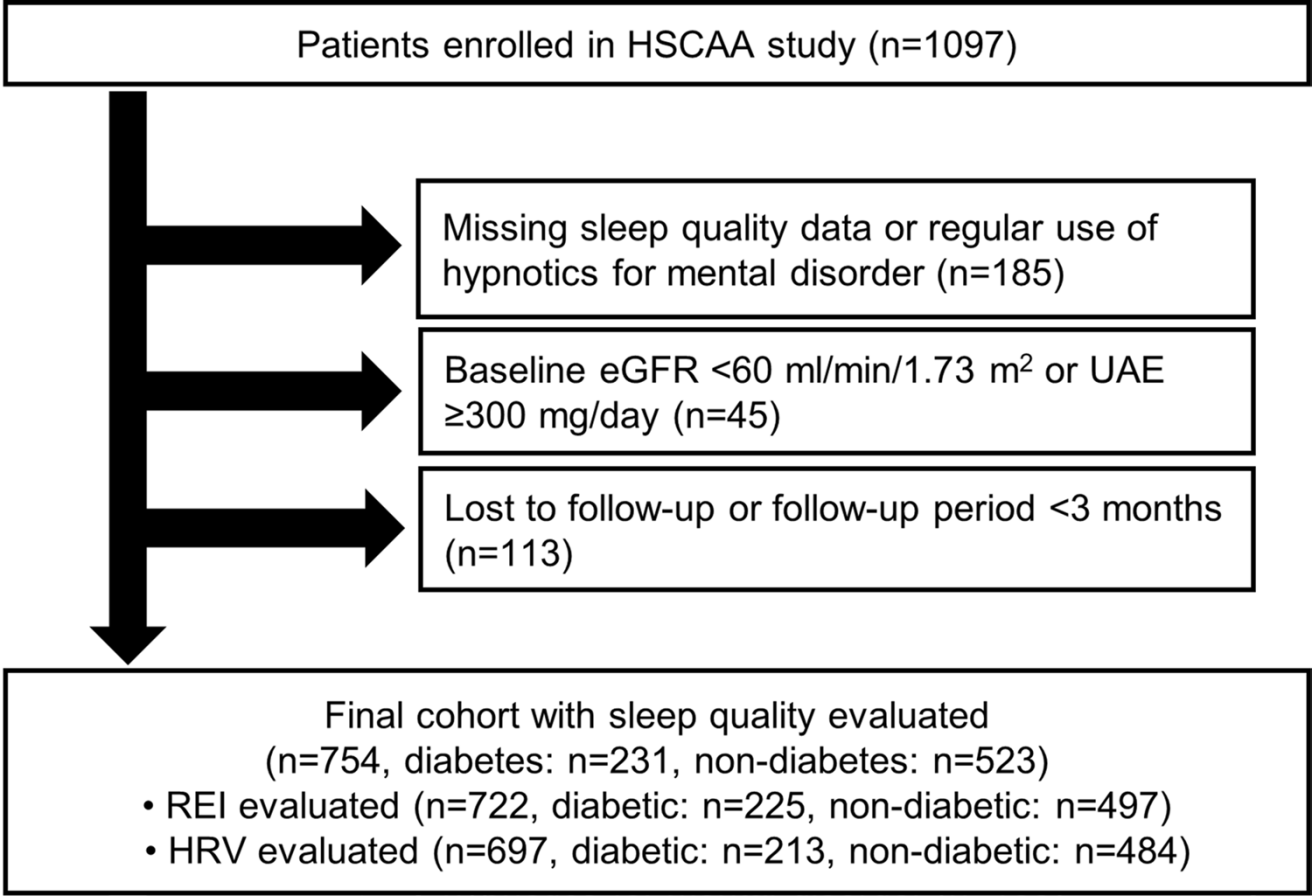
Flowchart of criteria for selecting study participants. *HSCAA* hyogo sleep cardio-autonomic atherosclerosis, *eGFR* estimated glomerular filtration rate, *UAE* urinary albumin excretion, *REI* respiratory event index, *HRV* heart rate variability.

The ongoing HSCAA cohort study was started in October 2010, with 1097 patients registered up to March 2019 included in the present investigation. The study was approved by the Ethics Committee of Hyogo College of Medicine (approval No. 2351). All methods were conducted in accordance with relevant guidelines and regulations after written informed consent was obtained from all subjects. Sleep apnea, sleep quality, and autonomic nervous function were collectively evaluated at the baseline, then enrolled patients were routinely followed until June 2019. Among the factors examined, serum creatinine concentration was determined every 3 months and eGFR was calculated at each examination. After excluding 185 patients with missing data for sleep quality or regular use of hypnotics for mental disorders, 45 patients with baseline eGFR < 60 ml/min/1.73 m^2^ or urinary albumin excretion ≥ 300 mg/day, and 113 lost to follow-up or with a follow-up period < 3 months, baseline and follow-up data of the remaining 754 (diabetes: n = 231, non-diabetes: n = 523) were used for the present analyses (Fig. 1). Among the enrolled patients, 722 were evaluated for respiratory event index (REI) (diabetes: n = 225, non-diabetes: n = 497) and 697 for heart rate variability (HRV) (diabetes: n = 213, non-diabetes: n = 484).

### Classical cardiovascular risk factors

8 type 1 and 223 type 2 diabetes patients were included in the diabetic group. Type 2 diabetes was diagnosed based on fasting plasma glucose ≥ 126 mg/dl, causal plasma glucose ≥ 200 mg/dl, or 2-h plasma glucose ≥ 200 mg/dl shown by a 75-g oral glucose tolerance test, or history of therapy for diabetes^10^. Type 1 diabetes was defined by typical clinical history findings, such as rapid onset of ketoacidosis, absolute deficiency of insulin secretion following treatment with multiple doses of insulin, or positivity for the autoantibody against glutamic acid decarboxylase. We defined history of cardiovascular events as past experience with coronary heart disease (myocardial infarction, coronary intervention) or stroke. Hypertension was defined as systolic blood pressure ≥ 140 mmHg, diastolic blood pressure ≥ 90 mmHg, or treatment for hypertension. Dyslipidemia was defined as low density lipoprotein cholesterol ≥ 140 mg/dl, high density lipoprotein cholesterol ≤ 40 mg/dl, triglyceride level ≥ 150 mg/dl, or presently under treatment for dyslipidemia^11^. Serum creatinine concentration was determined using an enzymatic method. eGFR in each patient was calculated using an equation for subjects in Japan, as follows: eGFR (ml/min/1.73 m^2^) = 194 × age(years)^-0.287^ × S-Cr^-1.094^ (if female, × 0.739)^12^. Urinary albumin was determined using an immunoturbidimetry method, with microalbuminuria defined as UAE ≥ 30 mg/day^13^.

### Sleep and autonomic nervous function

To examine the presence of sleep apnea, we used an Apnomonitor (SAS-2100^®^, Teijin, Tokyo, Japan) to determine REI, as previously described^14,15^. An actigraphy device (Ambulatory Monitoring, Inc., Ardley, New York, USA), which senses motion as acceleration, was placed on the wrist of the non-dominant arm and used for quantitative analysis of sleep quality, as previously reported^16–19^. Actigraphy results have been shown to be as reliable as those obtained with polysomnography for determination of sleep disturbance^20^. According to published recommendations for clinical use of actigraphy results^21^, activity index as a parameter of sleep quality was calculated as total body motion during sleep time, with a higher activity index value considered to be related to lower sleep quality.

HRV using an Active Tracer device (AC-301A^®^, Arm Electronics, Tokyo, Japan) was used for noninvasive determination of cardiac modulation based on autonomic balance, as previously described^14,22–24^. The final 24-h series of data from the 48-h recording was analyzed using a MemCalc Chiram 3 system, version 2.0 (Suwa Trust, Tokyo, Japan). Ectopic beats, noise data, and artifacts were manually corrected or excluded from calculation. According to the recommendations for clinical use of HRV^25^, the coefficient of variation of the R–R interval (CVRR) within the time domain was calculated.

### Study outcomes

For the present study, the primary renal outcome was defined as a decline in eGFR to less than 60 ml/min/1.73 m2 for more than 3 months, as previously recommended.

### Statistical analysis

The enrolled subjects were divided into the diabetic (n = 231) and non-diabetic (n = 523) groups. According to previously published studies of clinical use of actigraphy^21^ and apnomonitor^26^ results, they were also divided into those with and without PSQ using an activity index cut-off value of 50, and with and without sleep apnea using an REI cut-off value of 5. For examining autonomic nervous function, the subjects were divided into those with low and normal or high HRV based on the median CVRR value. To compare variables between the diabetic and non-diabetic groups, a non-repeated t-test (continuous variables with normal distribution), Mann–Whitney test (continuous variables with skewed distribution), and chi-squared test (categorical variables) were utilized as appropriate. The outcome rates for the groups were compared using Kaplan–Meier analysis and a log-rank test. Prognostic variables for decline in renal function were examined using a univariate or multivariate Cox proportional hazards regression model. All statistical analyses were performed using the Statistical Package for the Social Sciences software platform (PASW Statistics, version 18.0). All reported *p* values are two-tailed and were considered statistically significant at < 0.05.

## Results

### Baseline characteristics of study participant

Baseline characteristics of the subjects after dividing into those with and without diabetes are shown in Table 1. As compared to the non-diabetic group, patients with diabetes were older and exhibited greater values for body mass index (BMI), systolic or diastolic blood pressure (BP), albuminuria, HbA1c, fasting plasma glucose, activity index (median 33.5), and REI (median 8.9), and also showed a greater percentage of male gender, current smokers, subjects with a past history of cardiovascular disease (CVD), hypertension, or dyslipidemia, users of an Angiotensin converting enzyme (ACE) inhibitor or Angiotensin II receptor blocker (ARB), as well as cases of sleep apnea (66.6%) and low heart rate variability (HRV) (56.3%). The percentage of subjects with PSQ was not significantly different between the groups, while there were also no significant differences between them for the variables renal function, blood urea nitrogen, creatinine, and baseline eGFR (mean; DM: 89.8 ml/min/1.73m^2^, non-DM: 87.3 ml/min/1.73m^2^).

**Table 1:** Comparisons of baseline clinical characteristics between patients with and without diabetes.

Variables	Total	Diabetes	Non-diabetes	*p*
Number	754	231	523	
Age, years	57.7 ± 0.5	62.1 ± 0.7	55.8 ± 0.6	< 0.001
Male gender, n (%)	353 (46.8)	130 (56.3)	223 (42.6)	< 0.001
Body mass index, kg/m^2^	24.3 ± 0.1	25.5 ± 0.3	23.7 ± 0.2	< 0.001
Current smoking, n (%)	179 (23.7)	68 (29.4)	111 (21.2)	0.018
Past history of CVD, n (%)	94 (12.5)	45 (19.5)	49 (9.4)	< 0.001
Systolic BP, mmHg	115.2 ± 1.4	122.7 ± 2.0	111.8 ± 1.8	< 0.001
Diastolic BP, mmHg	69.0 ± 0.8	72.3 ± 1.2	67.4 ± 1.1	0.016
LDL-cholesterol, mg/dL	110.2 ± 2.0	102.4 ± 3.1	114.4 ± 2.5	< 0.011
HDL-cholesterol, mg/dL	55.9 ± 0.7	52.6 ± 1.0	57.6 ± 0.9	0.001
Triacylglycerol, mg/dL	129.9 ± 3.6	137.9 ± 7.3	125.6 ± 3.9	0.151
Uric acid, mg/dL	5.4 ± 0.0	5.4 ± 0.1	5.4 ± 0.0	0.590
Blood urea nitrogen, mg/dL	13.7 ± 0.1	14.1 ± 0.3	13.5 ± 0.1	0.066
Creatinine, mg/dL	0.65 ± 0.00	0.64 ± 0.00	0.65 ± 0.00	0.487
Baseline eGFR, ml/min/1.73m^2^	88.0 ± 0.7	89.8 ± 1.5	87.3 ± 0.8	0.133
Albuminuria, mg/gCr	5.9 (0.0–15.1)	10.2 (0.0–30.3)	4.7 (0.0–11.5)	< 0.001
Fasting plasma glucose, mg/dL	108.1 ± 1.2	134.1 ± 2.7	94.5 ± 0.6	< 0.001
HbA1c (%)	5.9 ± 0.0	7.2 ± 0.1	5.2 ± 0.0	< 0.001
**Comorbidities**				
Hypertension, n (%)	470 (62.3)	166 (71.9)	304 (58.1)	< 0.001
Dyslipidemia, n (%)	402 (53.3)	154 (66.7)	248 (47.4)	< 0.001
**Anti-hypertensive drugs**				
Calcium-channel blockers, n (%)	313 (41.5)	103 (44.6)	210 (40.2)	0.289
ACE inhibitor or ARB, n (%)	148 (19.6)	76 (32.9)	72 (13.8)	< 0.001
α or β blocker, n (%)	84 (11.1)	23 (10.0)	61 (11.7)	0.575
Diuretic agent, n (%)	40 (5.3)	18 (7.8)	22 (4.2)	0.064
Statin, n (%)	193 (25.6)	100 (43.3)	93 (17.8)	< 0.001
**Anti-diabetic drugs**				
DPP-4 inhibitor, n (%)	–	79 (34.1)	–	N/A
GLP-1 analogue, n (%)	–	12 (5.1)	–	N/A
Sulfonylurea, n (%)	–	49 (21.3)	–	N/A
Thiazolidine, n (%)	–	18 (3.5)	–	N/A
Metformin, n (%)	–	75 (32.5)	–	N/A
SGLT2 inhibitor, n (%)	–	2 (0.9)	–	N/A
Insulin, n (%)	–	45 (19.6)	–	N/A
Diabetes duration, years	–	9.1 ± 0.6	–	N/A
**Diabetic microvascular complications**				
Neuropathy, n (%)	–	70 (30.3)	–	N/A
Retinopathy, n (%)	–	28 (12.1)	–	N/A
Nephropathy, n (%)	–	135 (58.4)	–	N/A
PSQ, n (%)	102 (13.5)	35 (15.2)	67 (12.8)	0.41
Activity index	30.5 (21.8–42.5)	33.5 (25.4–44.7)	29.7 (20.4–41.1)	0.003
Number	722	225	497	–
Sleep apnea, n (%)	373 (51.7)	150 (66.6)	223 (44.8)	< 0.001
REI	5.6 (1.4–12.9)	8.9 (3.2–17.9)	4.0 (0.9–11.0)	< 0.001
Number	697	213	484	–
Low HRV, n (%)	348 (49.9)	120 (56.3)	228 (47.1)	0.039
CVRR	13.5 (11.4–16.1)	12.9 (11.2–15.5)	13.7 (11.5–16.3)	0.427

Data are presented as the mean ± standard error or median (25th–75th percentile) for continuous variables or number (%) for dichotomous variables. P values are shown for comparisons of mean (t-test) and median (Mann–Whitney test) values. Percentages were determined with a chi-squared test.

*CVD* cardiovascular disease, *BP* blood pressure, *LDL* low density lipoprotein, *HDL* high density lipoprotein, *eGFR* estimated glomerular filtration rate, *ACE* angiotensin converting enzyme, *ARB* angiotensin II receptor blocker, *DPP* dipeptidyl peptidase, *GLP* glucagon-like peptide, *PSQ* poor sleep quality, *REI* respiratory event index, *HRV* heart rate variability, *CVRR* coefficient of variation of R–R interval, *NA* not applicable.

### Association of sleep quality, sleep apnea and HRV with renal outcome

**Figure 2:**
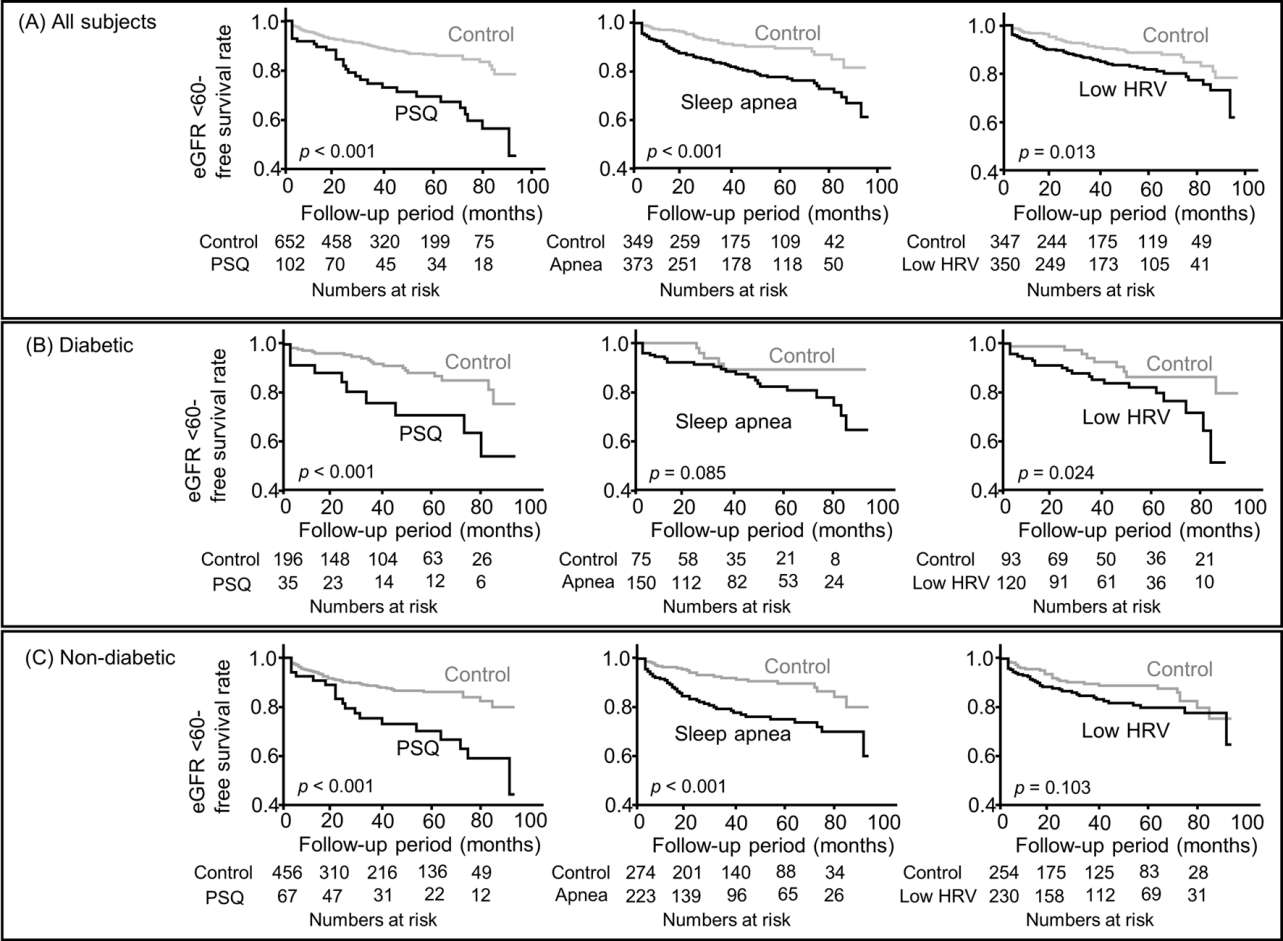
Kaplan–Meier analysis of associations of PSQ, sleep apnea, and autonomic nervous function with renal function decline in diabetic and non-diabetic patients. Subjects in the diabetic and non-diabetic groups were compared based on the presence or absence of PSQ, sleep apnea, and low HRV. Probability was analyzed using a log-rank test. *PSQ* poor sleep quality, *HRV* heart rate variability.

The median follow-up period of all analyzed subjects was 38.5 months. Kaplan–Meier analysis findings indicated that those with PSQ, sleep apnea, and low HRV exhibited a significantly greater risk for decline in renal function as compared to the others (Fig. 2A). Diabetic patients with PSQ and low HRV, but not sleep apnea, had a significantly greater risk for renal outcome as compared to the control group (log-rank test, PSQ; *p* < 0.001, low HRV; *p* = 0.024) (Fig. 2B). On the other hand, non-diabetic patients with PSQ and sleep apnea had a significantly greater risk for renal outcome, but not those with low HRV (Fig. 2C).

Results of univariate Cox proportional hazards regression analysis (Table 2) showed that age, baseline eGFR, and presence of albuminuria were significantly associated with renal outcome in both the diabetic and non-diabetic groups. In the patients with diabetes, use of anti-diuretics, and diabetes duration were significantly associated with higher risk for renal outcome, while past history of cardiovascular disease, hypertension, use of calcium channel blockers or α/β blockers, and use of statin were significantly associated with higher risk in the non-diabetic group. PSQ and low HRV were significantly associated with renal outcome in the diabetic group [PSQ: hazard ratio (HR) 2.90; 95% confidence interval (CI) 1.36–6.18, *p* = 0.005; low HRV: HR 2.43, 95% CI 1.09–5.41, *p* = 0.029], while sleep apnea was not. In the non-diabetic group, PSQ and sleep apnea, but not low HRV, were significantly associated with renal outcome (PSQ: HR 2.40, 95% CI 1.43–4.03, *p* < 0.001; sleep apnea: HR 2.54, 95% CI 1.56–4.13, *p* < 0.001).

**Table 2:** Univariate Cox proportional analyses of the factors associated with renal outcome.

Variables	Diabetes		Non-diabetes	
	HR (95% CI)	*p*	HR (95% CI)	*p*
Age (per 10-year period)	1.46 (1.02–2.07)	0.034	1.83 (1.49–2.25)	< 0.001
Gender (female = 0, male = 1)	0.83 (0.41–1.70)	0.619	1.56 (0.99–2.48)	0.054
Body mass index (< 25 kg/m^2^ = 0, ≥ 25 kg/m^2^ = 1)	1.26 (0.62–2.56)	0.515	0.90 (0.54–1.48)	0.680
Current smoking (no = 0, yes = 1)	0.61 (0.25–1.50)	0.285	1.09 (0.63–1.88)	0.748
Past history of CVD (no = 0, yes = 1)	1.83 (0.86–3.89)	0.114	2.15 (1.15–4.00)	0.015
Systolic BP	1.02 (1.00–1.04)	0.081	0.99 (0.99–1.00)	0.701
Diastolic BP	1.01 (0.98–1.04)	0.622	0.99 (0.98–1.00)	0.183
LDL-cholesterol	1.01 (0.99–1.02)	0.119	1.00 (0.99–1.01)	0.295
HDL-cholesterol	1.00 (0.97–1.02)	0.951	0.98 (0.97–1.00)	0.147
Triacylglycerol	0.99 (0.99–1.00)	0.738	1.00 (0.99–1.00)	0.585
Uric acid	1.04 (0.82–1.33)	0.720	1.08 (0.90–1.28)	0.395
Baseline eGFR (per 5 ml/min/1.73 m^2^)	0.80 (0.70–0.91)	< 0.001	0.56 (0.48–0.64)	< 0.001
Albuminuria (< 30 mg/gCr = 0, ≥ 30 mg/gCr = 1)	2.67 (1.31–5.42)	0.006	3.56 (2.06–6.14)	< 0.001
Fasting plasma glucose	1.00 (0.99–1.01)	0.941	1.01 (0.99–1.03)	0.281
HbA1c	0.99 (0.76–1.28)	0.959	1.24 (0.61–2.52)	0.546
Hypertension (no = 0, yes = 1)	1.80 (0.73–4.40)	0.196	3.47 (1.94–6.22)	< 0.001
Dyslipidemia (no = 0, yes = 1)	1.13 (0.53–2.40)	0.748	1.42 (0.89–2.25)	0.137
**Anti-hypertensive drugs**				
Calcium-channel blockers (no = 0, yes = 1)	2.04 (0.99–4.22)	0.052	2.41 (1.51–3.85)	< 0.001
ACE inhibitor or ARB (no = 0, yes = 1)	1.79 (0.88–3.65)	0.104	1.65 (0.94–2.88)	0.077
α or β blocker (no = 0, yes = 1)	1.86 (0.65–5.35)	0.246	2.54 (1.49–4.33)	< 0.001
Diuretic agent (no = 0, yes = 1)	3.65 (1.49–8.94)	0.004	1.64 (0.66–4.07)	0.285
Statin (no = 0, yes = 1)	1.86 (0.90–3.84)	0.091	2.17 (1.33–3.54)	0.001
**Anti-diabetic drugs**				
DPP-4 inhibitor (no = 0, yes = 1)	0.87 (0.41–1.85)	0.725	N/A	
GLP-1 analogue (no = 0, yes = 1)	2.43 (0.73–8.06)	0.144	N/A	
Sulfonylurea (no = 0, yes = 1)	0.51 (0.18–1.47)	0.217	N/A	
Thiazolidine (no = 0, yes = 1)	0.66 (0.15–2.81)	0.582	N/A	
Metformin (no = 0, yes = 1)	1.45 (0.71–2.97)	0.305	N/A	
SGLT2 inhibitor (no = 0, yes = 1)	N/A		N/A	
Insulin (no = 0, yes = 1)	1.47 (0.65–3.28)	0.348	N/A	
Diabetes duration	1.05 (1.01–1.09)	0.006	N/A	
**Diabetic microvascular complications**				
Neuropathy (no = 0, yes = 1)	1.35 (0.66–2.75)	0.401	N/A	
Retinopathy (no = 0, yes = 1)	2.10 (0.89–4.91)	0.086	N/A	
Nephropathy (no = 0, yes = 1)	2.11 (1.05–4.24)	0.035	N/A	
PSQ (no = 0, yes = 1)	2.90 (1.36–6.18)	0.005	2.40 (1.43–4.03)	< 0.001
Sleep apnea (no = 0, yes = 1)	2.26 (0.86–5.92)	0.094	2.54 (1.56–4.13)	< 0.001
Low HRV (no = 0, yes = 1)	2.43 (1.09–5.41)	0.029	1.49 (0.91–2.43)	0.106

*DM* diabetes mellitus, *CVD* cardiovascular disease, *BP* blood pressure, *LDL* low density lipoprotein, *HDL* high density lipoprotein, *eGFR* estimated glomerular filtration rate, *ACE* angiotensin converting enzyme, *ARB* angiotensin II receptor blocker, *DPP* dipeptidyl peptidase, *GLP* glucagon-like peptide, *PSQ* poor sleep quality, *HRV* heart rate variability, *NA* not applicable, *HR* hazard ratio, *CI* confidence interval.

To further examine whether sleep quality, sleep apnea, and low HRV had independent associations with renal outcome, multivariate Cox proportional analysis was performed (Table 3). In models that included age, gender, body mass index, smoking status, history of cardiovascular disease, hypertension, dyslipidemia, HbA1c, baseline eGFR, and albuminuria, PSQ showed a borderline significant association with decline in renal function in the diabetic group (HR 2.15, 95% CI 0.88–5.23, *p* = 0.091) (Model 1). As for the non-diabetic group, that model indicated that PSQ was significantly associated with renal outcome (HR 2.20, 95% CI 1.09–4.40, *p* = 0.025). Sleep apnea was not significantly associated with renal outcome after adjustment with other clinical factors in either the diabetic or non-diabetic group (Model 2). Low HRV was significantly and independently associated with renal function decline in patients with diabetes (HR 2.40, 95% CI 1.02–5.60, *p* = 0.043), but not in those without diabetes (Model 3). Of particular interest, the association of PSQ with renal outcome was significant after further adjustments for low HRV with (Model 7) or without (Model 5) sleep apnea in the diabetic group. In the non-diabetic group, the association of PSQ with renal outcome remained significant even after further adjustments for sleep apnea (Model 4), low HRV (Model 5), and both (Model 7). Also, the significant association of low HRV with renal outcome in the diabetic subjects was independent of PSQ (Model 5), while it was partially attenuated by sleep apnea (Models 6, 7).

**Table 3:** Multivariate Cox proportional analysis of factors associated with decline in renal function.

Variables	Diabetes		Non-diabetes	
	HR (95% CI)	*p*	HR (95% CI)	*p*
**Model 1**				
PSQ (no = 0, yes = 1)	2.15 (0.88–5.23)	0.091	2.20 (1.09–4.40)	0.025
**Model 2**				
Sleep apnea (no = 0, yes = 1)	1.58 (0.51–4.92)	0.422	1.36 (0.70–2.63)	0.358
**Model 3**				
Low HRV (no = 0, yes = 1)	2.40 (1.02–5.60)	0.043	0.81 (0.44–1.51)	0.525
**Model 4**				
PSQ (no = 0, yes = 1)	2.10 (0.84–5.23)	0.110	2.38 (1.17–4.82)	0.015
Sleep apnea (no = 0, yes = 1)	1.54 (0.49–4.81)	0.457	1.24 (0.63–2.43)	0.527
**Model 5**				
PSQ (no = 0, yes = 1)	2.60 (1.05–6.43)	0.038	2.37 (1.15–4.86)	0.018
Low HRV (no = 0, yes = 1)	2.60 (1.09–6.16)	0.029	0.79 (0.42–1.48)	0.470
**Model 6**				
Sleep apnea (no = 0, yes = 1)	1.54 (0.49–4.79)	0.453	1.56 (0.77–3.16)	0.216
Low HRV (no = 0, yes = 1)	2.09 (0.87–4.97)	0.095	0.76 (0.41–1.43)	0.411
**Model 7**				
PSQ (no = 0, yes = 1)	2.57 (1.01–6.53)	0.045	2.33 (1.12–4.84)	0.022
Sleep apnea (no = 0, yes = 1)	1.57 (0.49–5.03)	0.442	1.43 (0.69–2.94)	0.329
Low HRV (no = 0, yes = 1)	2.27 (0.94–5.47)	0.068	0.75 (0.39–1.42)	0.380

In all models used for multivariate Cox proportional hazards analysis, covariates included age, male gender, body mass index, current smoking, history of CVD, hypertension, dyslipidemia, HbA1c, baseline eGFR, and albuminuria. Model 1 included the presence of PSQ, Model 2 sleep apnea, Model 3 low HRV, Model 4 PSQ and sleep apnea, Model 5 PSQ and low HRV, Model 6 sleep apnea and low HRV, Model 7 PSQ, sleep apnea, and low HRV, in addition to the other covariates mentioned.

*CVD* cardiovascular disease, *eGFR* estimated glomerular filtration rate, *PSQ* poor sleep quality, *HRV* heart rate variability, *HR* hazard ratio, *CI* confidence interval.

## Discussion

This is the first known study to examine the integrated impacts of sleep apnea, sleep quality, and autonomic function as predictors of decline in renal function in diabetes and non-diabetes patients without CKD. The results show that PSQ and low HRV are important predictors for decline in renal function in diabetic patients without CKD, with those relationships independent of known classical risk factors. In contrast, in the present non-diabetic subjects, PSQ, but not low HRV, was an independent predictor for decline in renal function.

In previous studies, sleep quality was shown to be associated with a higher risk for renal function decline^2,27^. However, no known investigation has been conducted to examine risk using quantitative measurements of sleep quality, and compare between diabetic and non-diabetic patients without CKD. The present results clearly showed that PSQ was indeed an essential predictor for decline in renal function in both groups. Among the causative factors for PSQ, sleep apnea has been shown to be strongly associated with renal dysfunction^4,5,26^, though a conflicting report has been presented^28^. Tahrani et al. reported that eGFR decreased faster in T2D patients with OSA compared to similar patients without OSA after an average of 2.5 years of follow-up^26^. In our diabetic subjects, although those with PSQ exhibited significantly higher REI values (median: 14.5) than those without PSQ (median: 8.1), sleep apnea was not independently associated with decline in renal function in the diabetic group in multivariate Cox proportional hazards analyses. This discrepancy may be the results of differences in lower BMI (25.5 vs. 35.4) and lower HbA1c (7.2 vs. 8.3) in our patients with sleep apnea, both of which are well-known risk factors related to renal dysfunction. Additionally, other covariates included only in our study, such as baseline eGFR and albuminuria values, or statistical methods, Cox proportional vs. logistic regression analyses may account for the differences. A retrospective study conducted in Taiwan found a relationship of sleep quality with incidence of CKD in subjects without apnea^29^. In the present study, the association of PSQ with renal outcome was shown to be unaffected after adjustment for sleep apnea in both diabetic and non-diabetic patients. Thus, the predictive impact of PSQ on progression of renal dysfunction appears necessarily not the result of potentially coexisting sleep apnea.

The present results are the first to show that low HRV is an important predictor for decline in renal function in pre-CKD patients with diabetes, but not in those without diabetes. Furthermore, that relationship was found to be independent of classical risk factors as well as sleep quality or presence of apnea. In the Atherosclerosis Risk in Communities (ARIC) study, which had a median follow-up period of 16 years, low HRV was demonstrated to be associated with occurrence of ESRD- and CKD-related hospitalization in CKD patients^30^, though they did not investigate the predictive value of low HRV in diabetic patients by subgroup analysis. The present findings are the first to show that the impact of autonomic function on renal function decline is dependent on the presence of diabetes. It is not clear at present why low HRV is associated with higher risk for renal dysfunction only in diabetic patients during the pre-CKD phase. Nevertheless, it is considered that the effects of low HRV may not be dependent on the presence of diabetic neuropathy, since that was not significantly associated with decline in renal function in the present subjects. Autonomic nervous imbalance has been speculated to have potential to mediate the effect of sleep on renal function^7,31^. In our previous investigations, sleep disturbance was shown to be associated with autonomic dysfunction and fluctuations in nocturnal blood pressure^23,32^. Uncontrolled nighttime sympathetic activation due to decreased autonomic function might be potentially involved, at least in part, in the relationship of PSQ with renal function decline. However, the present results clearly demonstrated a significant association of PSQ with decline in renal function, even after adjustments for low HRV along with well-established renal risk factors. Potential candidates include brain-derived neurotrophic factor (BDNF), which has been reported to have critical roles in survival, growth, and maintenance, as well as death of central and peripheral neurons^33,34^, while more recent studies including ours have also demonstrated associations of BDNF with sleep disturbance and autonomic function^13,35,36^. In that previous study conducted by our group, positive and significant associations of plasma BDNF with autonomic function and nighttime blood pressure fluctuation were found^32^, while our more recent report noted that low plasma BDNF level was independently associated with decline in renal function^13^.

The present study has some limitations. First, reduced eGFR (less than 60 ml/min/1.73 m^2^) for more than 3 months was used as the primary clinical endpoint for patients without CKD, whereas another well-used endpoint, 30% decrease in eGFR, was not, because the number of events during the study period were not adequate for analysis. Second, 24-h urinary albumin monitoring was not performed during the follow-up period, thus only decreased eGFR was used as the primary endpoint. Third, though our findings suggest that objective sleep quality and autonomic imbalance are important predictors of decline in renal function in DM patients in the pre-CKD phase, a well-controlled randomized study will be necessary to establish that causal relationship. Fourth, information of menopause age was not collected in our study, even though the relationship between menopause and sleep apnea has long been pointed out^37^. Indeed, REI was significantly higher in female over 50 years than those under 50. However, renal outcome was not significantly different between female with > 50 years and those with < 50 years of age (data not shown). Fifth, there may be misdiagnosed patients in the no sleep apnea group because REI values tend to be lower than AHI values measured by polysomnography^15^. Finally, while potential causative factors related to the association of PSQ with decline in renal function were extensively examined, the underlying detailed mechanisms for the relationship were not identified. Nevertheless, to the best of our knowledge, no previous study has explored the integrated impact of sleep quality, sleep apnea, and autonomic balance on renal function using comparisons of patients with and without diabetes.

In conclusion, PSQ and low HRV independent of sleep apnea are important risk factors for decline in renal function in pre-CKD diabetic patients.
